# Molecular Epidemiology of Norovirus (NoV) Infection in Mie Prefecture: The Kinetics of Norovirus Antigenemia in Pediatric Patients

**DOI:** 10.3390/v14020173

**Published:** 2022-01-18

**Authors:** Jennifer X. Amexo, Manami Negoro, Elijah Deku-Mwin Kuurdor, Belinda L. Lartey, Shigeru Sokejima, Ken Sugata, Prince Baffour Tonto, Kiyosu Taniguchi

**Affiliations:** 1 Department of Public Health and Occupational Medicine, Mie University Graduate School of Medicine, 2-174 Edobashi, Tsu-shi 514-8507, Japan; kuurdore@yahoo.com (E.D.-M.K.); sokejima@med.mie-u.ac.jp (S.S.); 2 Department of Clinical Research, National Hospital Organization, Mie National Hospital, Tsu-shi 514-0125, Japan; negoro.manami.zt@mail.hosp.go.jp (M.N.); ksugata45@gmail.com (K.S.); princebaffourtonto@gmail.com (P.B.T.); 3 Department of Electron Microscopy and Histopathology, Noguchi Memorial Institute for Medical Research, College of Health Sciences, University of Ghana, Accra 00233, Ghana; blartey@noguchi.ug.edu.gh; 4 Epidemiology Centre for Disease Control and Prevention, Mie University Hospital, Tsu-shi 514-8507, Japan

**Keywords:** antigenemia, ELISA, real-time RT-PCR, norovirus, RNAemia

## Abstract

Few studies have shown the presence of norovirus (NoV) RNA in blood circulation but there is no data on norovirus antigenemia. We examined both antigenemia and RNAemia from the sera of children with NoV infections and studied whether norovirus antigenemia is correlated with the levels of norovirus-specific antibodies and clinical severity of gastroenteritis. Both stool and serum samples were collected from 63 children admitted to Mie National Hospital with acute NoV gastroenteritis. Norovirus antigen and RNA were detected in sera by ELISA and real-time RT-PCR, respectively. NoV antigenemia was found in 54.8% (34/62) and RNAemia in 14.3% (9/63) of sera samples. Antigenemia was more common in the younger age group (0–2 years) than in the older age groups, and most patients were male. There was no correlation between stool viral load and norovirus antigen (NoV-Ag) levels (r_s_ = −0.063; Cl −0.3150 to 0.1967; *p* = 0.6251). Higher levels of acute norovirus-specific IgG serum antibodies resulted in a lower antigenemia OD value (*n* = 61; r = −0.4258; CI −0.62 to −0.19; *p* = 0.0006). Norovirus antigenemia occurred more commonly in children under 2 years of age with NoV-associated acute gastroenteritis. The occurrence of antigenemia was not correlated with stool viral load or disease severity.

## 1. Introduction

Norovirus (NoV) is one of the leading causes of acute gastroenteritis (AGE) globally, causing both community-acquired and healthcare-associated outbreaks [1]. Noroviruses are single-stranded RNA viruses that are classified into ten genogroups (GI–GX) [2]. GI and GII have been implicated in most human infections [3]. For more than a decade, GII.4 has remained the predominant strain associated with major outbreaks worldwide [4]. Studies have shown that GII.4-associated diseases are accompanied by more severe symptoms compared with other genotypes [5].

The modes of transmission for norovirus are through the fecal–oral route, via direct person-to-person transmission, and through contaminated food or water. All age ranges are affected, with children experiencing the highest incidence. Severe outcomes, including hospitalization and death, occur primarily in children and the elderly [3,6]. The prevalence of NoV seropositivity increases rapidly before the age of 5 years, when the majority of the population has been exposed to the virus [7].

Norovirus virus-like particles (NoV VLPs) are used for studying immune responses against NoV. The expression of the norovirus capsid VP1 protein results in the formation of VLPs that are morphologically and antigenically similar to its endemic virions [8].

Norovirus is shed from infected cells into the intestinal lumen, but NoV-RNA can also be detected in serum and cerebrospinal fluid [6]. Improvements in methods for detecting norovirus have led to numerous reports of extraintestinal manifestations, other than gastroenteritis associated with NoV [9,10,11].

While viral gastroenteritis is generally confined to the intestines, rotavirus and norovirus can result in antigenemia and the presence of nucleic acid in the blood of ill patients without any extraintestinal infections [12,13]. Few studies have focused on norovirus RNA and we were unable to find any studies on antigenemia let alone its importance. In this study, we sought to determine whether the levels of NoV antigen in serum are correlated with the levels of norovirus-specific antibodies and clinical severity in NoV antigenemia patients. In addition, cross-reactivity of NoV antibodies between the predominant GII.4 and GII.17 Kawasaki 308 (predominant in winter 2014–2015) genotypes was investigated.

The determination of cross-reactivity allowed ascertaining whether patients may have pre-existing antibodies resulting from a strain that was predominant in the past.

## 2. Materials and Methods

### 2.1. Sample Collection

The study was conducted at Mie National Hospital, Tsu, Japan, from November 2018 to March 2021. Beginning in November 2018, stool and serum samples for clinical assessment were taken from hospitalized patients suspected of acute gastroenteritis, stored at −80 °C, and later tested for norovirus after receiving informed consent. From the stored samples, we detected NoV from stool specimens of 293 cases of acute gastroenteritis; of these, 63 NoV cases had both stool and serum samples available for use in this study (Figure S1). Moreover, cases were identified in the database, and their clinical information was extracted from the electronic medical chart system by a supervising clinician and confirmed for clinical cases. This included children between the ages of 0 years to 18 years. The study protocol was approved by the Ethical Review Board of Mie National Hospital (12/2020). As part of the clinical assessment, rotavirus and adenovirus were tested using the rapid antigen test kit [14]. In cases negative for enteric viruses, a stool culture was also performed for enteric pathogens.

### 2.2. Norovirus Detection in Stool and Serum

Viral RNA was extracted using the QIAamp^®^ Viral RNA Minikit (Qiagen, Hilden, Germany) according to the manufacturer’s instructions. Complementary DNA (cDNA) synthesis was conducted using SuperScript II Reverse Transcriptase for real-time quantitative polymerase chain reaction (RT-qPCR) (Table S2). For the detection of norovirus GI, primers COG1F and COG1R and probes RING1-TP(a) and RING1-TP(b) were used. For NoV GII, primers COG2F/ALPF and COG2R and probe RING2AL-Tb [15] were used in a StepOnePlus™ real-time PCR system (Applied Biosystem, Thermo Fisher Scientific, Foster City, California, CA, USA). The viral load was estimated in terms of PCR cycle threshold C_T_ at which the amplicon was detected.

### 2.3. Norovirus Genomic Amplification and Genotyping

To analyze the genotype of the detected noroviruses, the capsid region C of ORF2 was amplified using the primer sets COG2F/G2SKR (first round) and G2SKF/G2SKR (second round), generating amplicons of ~387 and ~344 bp, respectively, for NoV GII [16]. For NoV GI, primers COG1F/G1SKR (first round) and G1SKF/G1SKR (second round) generated amplicons of ~381 and ~330 bp, respectively, all in a semi-nested PCR. In addition, we used the MON431/G2SKR that targets the ORF1/2 junction (~570 bp) in a single reaction. PCR was carried out using the TaKaRa Ex Taq^®^ Hot Start Version. The PCR conditions were as follows: 94 °C for 3 min; 40 cycles of PCR at 94 °C for 30 s, 55 °C for 30 s, and 72 °C for 1 min; and a final incubation at 72 °C for 15 min. After amplification, the PCR products were visualized by gel electrophoresis. Positive norovirus samples were prepared as DNA and primer premixed templates and sent to Eurofins genomics for sequencing (Eurofins Genomics Co., Ltd., Ota City, Tokyo).

### 2.4. Detection of Norovirus Antigen in Serum

Norovirus antigen was detected in serum using the commercial Human Norovirus Antigen ELISA kit (MyBioresource, San Diego, Southern California, CA, USA), according to the manufacturer’s instructions.

### 2.5. Serum Antibody ELISA

Norovirus virus-like particles (VLPs) were used as antigens. The GII.4 VLP and GII.17 VLP were kindly provided by Prof. Kazuhiko Kaytayama, Kitasato University, in a baculovirus expression system as described by Hansman et al. [17]. These VLPs were produced by a recombinant baculovirus expression system using the GII.4 Sydney 2012 variant named Ni 1315 and GII.17 Kawasaki-308 (accession number LC037415). Briefly, the GII.4 Ni1315 strain capsid VP1 sequence originated from a patient sample collected in 2013 in Niigata Prefecture, Japan. A recombinant NoV VP1 capsid protein was expressed in the insect cell line High Five cells (Invitrogen, Carlsbad, California, CA, USA), and VLPs secreted into the cell medium were collected by ultracentrifugation at 100,000× *g* in an SW32 rotor (Beckman, Palo Alto, California, CA, USA). VLPs were purified by CsCl equilibrium gradient ultracentrifugation. Purified VLPs were applied to a carbon-coated electron microscopy grid, stained with 2% uranyl acetate, and examined and their identity verified by electron microscopy. Sera were tested for anti-GII.4 and anti-GII.17 IgG by ELISA, as previously described in [18,19], with some modifications. Serum samples were diluted two-fold starting at 1:100 and plated on GII.4 Ni 1315 and GII.17 Kawasaki-308 VLP-coated (0.1 μg/mL and 0.5 μg/mL phosphate-buffered saline (PBS)) 96-well half-area microtiter plates blocked with 5% skimmed milk. One known positive sample and one known negative sample (5% skimmed milk in PBS-T) were included in every plate as controls. Serum dilutions were incubated on the plates for 1 h at room temperature. The bound antibodies were detected with goat anti-human IgG-HRP (Invitrogen, California, CA, USA), followed by a KPL TMB 2-component microwell peroxidase substrate system; reactions were stopped with 1 N hydrochloric acid. All washes were performed with 0.1% Tween 20 in 1× phosphate-buffered saline (PBS). The optical density (OD) was measured at 450 nm using the victor^2^ 1420 Multilabel counter (Wallac, Perkin Elmer, Turku, Finland) plate reader. The cut-off value was determined as two or three times the background signal.

### 2.6. Phylogenetic Analyses

Using the Basic Local Alignment Search Tool (BLAST) available on the NCBI website (https://blast.ncbi.nlm.nih.gov/Blast.cgi, accessed on 1 December 2021), reference sequences for phylogenetic analysis were retrieved. Multiple sequence alignment was carried out in Bioedit and MEGA-X software, and the best-fit nucleotide sequence substitution model was determined using MEGA-X software [20]. Phylogenetic trees were constructed using the Maximum Likelihood algorithm with 1000 bootstrap replicates and a General Time Reversible plus Invariant site (GTR + I). Sequences derived in this study were deposited in GenBank under the accession numbers OK103809-OK103830, OK103833-OK103836, and OK103839-OK103840.

### 2.7. Statistical Analyses

All data were entered into Microsoft Access and Excel Windows Version 2020 (Microsoft Corporation, Redmond, WA, USA), and analyses were performed using GraphPad Prism 9 and IBM SPSS 27. Differences between groups were analyzed using Fisher’s exact test (two-tailed) for categorical variables and Kruskal–Wallis test for differences in the antibody OD between the age groups. The Spearman correlation coefficient was used to determine the correlation between GII.4-specific antibodies and the serum antigen OD as well as the correlation between NoV-positive stool and antigenemia. Statistical significance was defined as *p* < 0.05.

## 3. Results

### 3.1. Detection of Norovirus RNA and Antigen in Serum

A total of 63 paired stool and serum samples were obtained from children reporting to the pediatric ward of Mie National Hospital with acute gastroenteritis. Of the 63 samples tested, the serum of one sample was completely exhausted after detection of NoV-RNA. Norovirus antigens (NoV antigenemia) were detected in 54.8% (34/62) by ELISA, whereas NoV-RNAs were detected in 14.3% (9/63) after RT-qPCR. Of the nine positive serum samples, five cases were not sequenced due to their low cDNA concentration.

There was also the presence of concomitant infection between norovirus and other pathogens (Table S1).

#### Estimation of NoV-RNA

**Figure 1:**
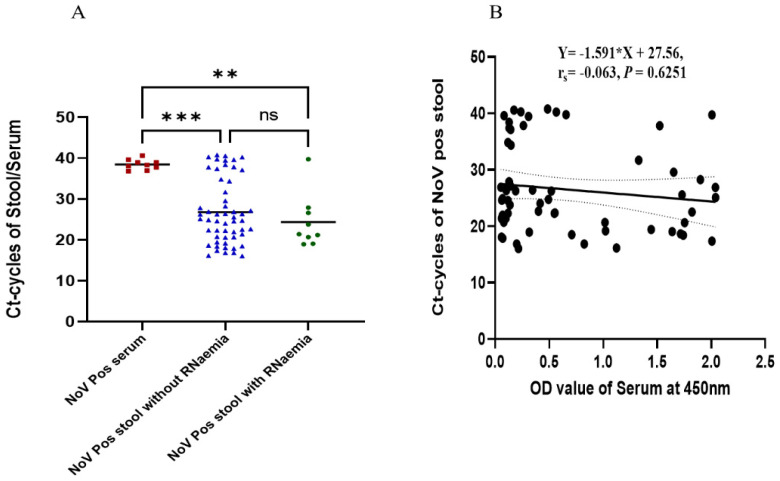
Viral load estimation from RT-qPCR and its correlation with NoV-Ag levels in serum and stool samples. (**A**) The red square dots represent positive serum samples, the dotted blue triangles represent positive stool samples without RNA present in their serum, and the circular green dots represent positive stool samples with RNA present in the serum. (**B**) Correlation between positive stool and antigenemia based on serum OD at 450 nm. ** *p* < 0.01, *** *p* < 0.001.

The mean Ct values detected in the stool sample of patients with NoV-positive and -negative sera were 24.37 (range 18.9–39.8) and 26.78 (range 16–40.8), respectively. This difference was, however, not statistically significant (Figure 1A) (*p* = 0.4991). As demonstrated in (Figure 1B), there was no correlation between the stool viral load and norovirus antigen (NoV-Ag) levels expressed as optical densities (r_s_ = −0.063; Cl −0.3150 to 0.1967; *p* = 0.6251, *n* = 62).

### 3.2. Clinical Features of NoV-Positive Cases

The age distribution of NoV-positive cases is shown in Table 1. Antigenemia was more often present in the younger age group (0–2 years) compared with the older age groups, and the majority of patients were males. There were no differences between the patients with antigenemia and patients without antigenemia with respect to the number of diarrheal stools, diarrhea duration and vomiting episodes. There was also no difference in transaminase levels, but CRP levels were higher in patients without antigenemia (Table 1). Children with NoV-Ag (as detected in serum) did not have more severe norovirus gastroenteritis episodes than those without NoV-Ag (Table 1).

### 3.3. Responses Due to Pre-Existing NoV Antibodies

**Figure 2:**
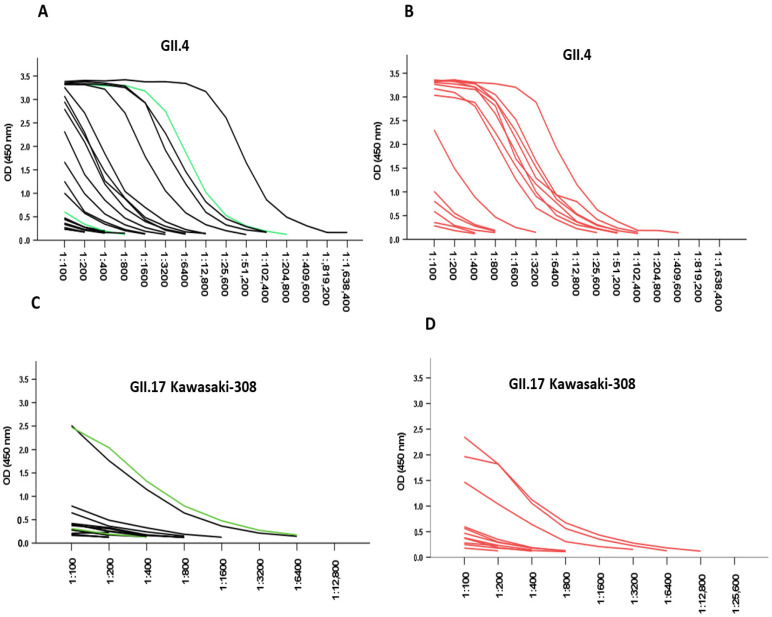
NoV IgG titration curves in serum samples from acute infections. (**A**) Sera from children with GII.4 Sydney/2012 (black lines, *n* = 21) and GI.2 genotype (green line, *n* = 2). (**B**) Other GII genotypes (red lines, *n* = 14) were titrated with two-fold serial dilutions, and IgG antibodies were analyzed against GII.4 Ni 1315 and GII.17 Kawasaki-308 genotypes. For GII.17 Kawasaki-308, (**C**) black lines, *n* = 17; green lines, *n* = 2; and (**D**) red lines, *n* = 12.

NoV-VLP-specific IgG antibody titration curves for sera from patients with acute infection are shown in Figure 2. Most of those infected with GII.4 Sydney/2012 (50.8%) had a high GII.4 Ni 1315 specific endpoint titer of ≤12,800 (Figure 2A), and all endpoint titers were ≤1,638,400. The same result was observed in those infected with other GII genotypes (GII.3, GII.2) as well as the G1.2 genotype (Figure 2B), and all endpoint titers were ≤409,600. Low endpoint titers (Figure 2C) were correspondingly obtained for GII.17 Kawasaki-308 in those infected with GII.4 Sydney/2012 as well as other genotypes (Figure 2D). There was also no significant difference in GII.4 Ni 1315 (*p* = 0.674) and GII.17 Kawasaki-308 (*p* = 0.068) specific titers in the acute phase between those infected with GII.4 Sydney/2012 and other genotypes.

**Figure 3:**
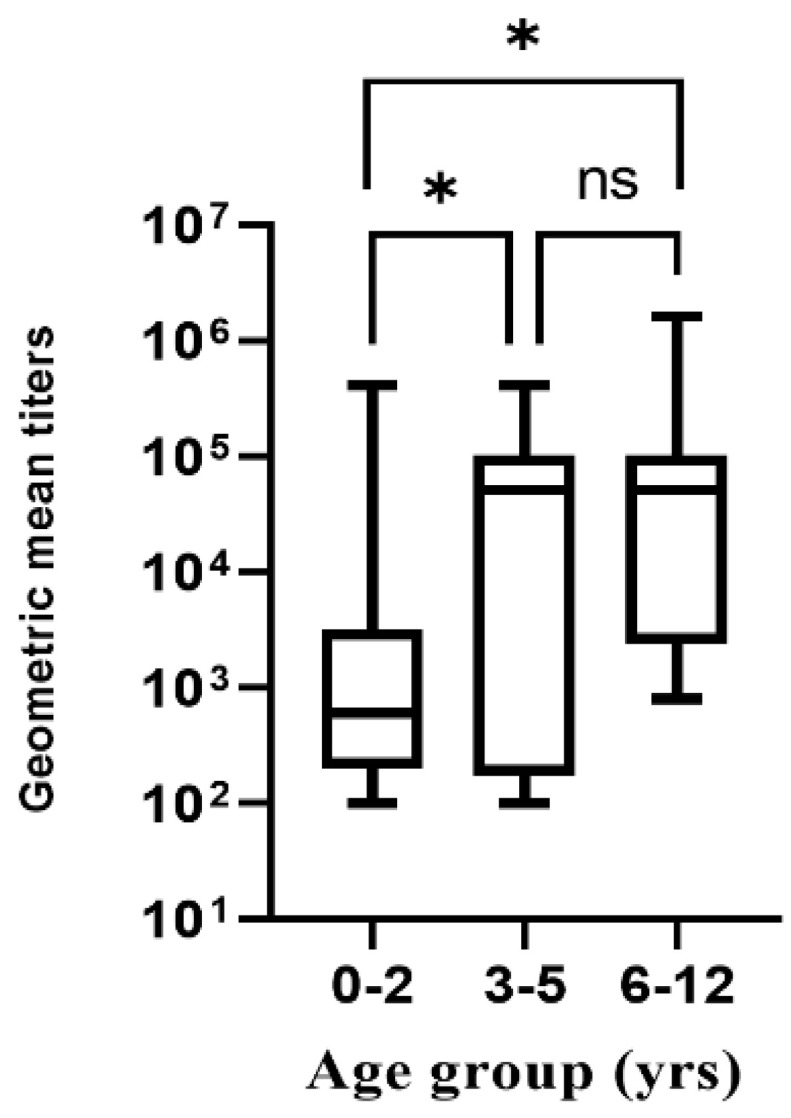
NoV pre-existing antibody titer responses for different age groups. * *p* < 0.05, ns: not significant.

Children in the age range of 0–2 years exhibited the lowest pre-existing antibody levels (median, 600), while those at 3–5 and 6–12 years had the highest pre-existing antibody level titers (median, 51,200 and 51,200, respectively) (0–2 vs. 3–5, *p =* 0.0476; 0–2 vs. 6–12, *p =* 0.0027) (Figure 3). Age was categorized into four groups: 0–2, 3–5, 6–12, and 13–18. The last age group had only one variable in the category and was therefore not included in the analysis for pre-existing NoV antibody titers.

### 3.4. Correlation of Antigenemia with Acute Antibody Titers

**Figure 4:**
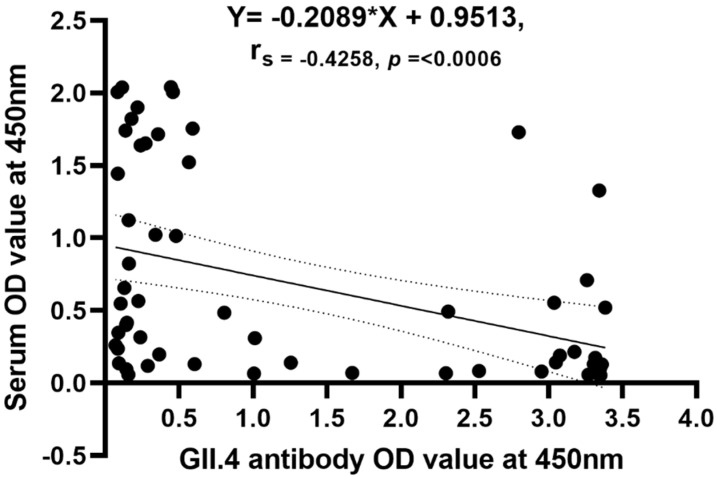
Correlation of antigenemia to serum acute antibody titers.

We tested whether higher levels of acute norovirus-specific IgG serum antibodies resulted in lower antigenemia OD values and observed a moderate negative correlation with the OD value from EIA (*n* = 61, r = −0.4258, CI; −0.62 to −0.19; *p* = 0.0006) (Figure 4).

### 3.5. Norovirus Genotypes and Phylogenetic Characteristics

**Figure 5:**
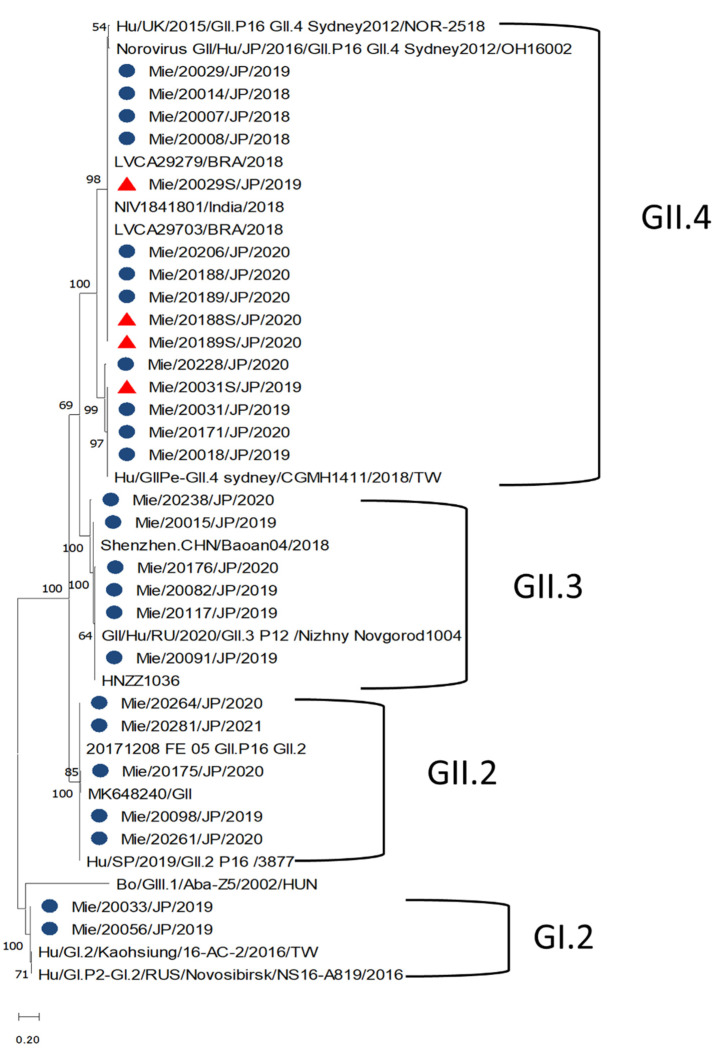
Phylogenetic analyses generated by the maximum likelihood test using the partial nucleotide sequences (338, 302, and 342 bp) from the C region of the capsid of norovirus. The strains reported in this study are indicated with a blue circle (stool) and a red triangle (sera).

Out of the 63 stool samples sequenced, the NoV genogroup GII was found in 54 cases and genogroup GI in 2 cases. However, seven cases were not sequenced because of the low concentration of cDNA. The genotypes detected from the stool samples were GII.4 (50.8%; 32/63), GII.3 (23.8%; 15/63), GII.2 (11.1%; 7/63), and GI.2 (3.2%; 2/63). The GII.4 had only one variant (Sydney/2012). Recombinant genotypes were also identified in this study, with GII.4 Sydney[P16] being the most predominant (*n* = 12) followed by GII.4 Sydney[P31] (*n* = 11), GII.3[P12] (*n* = 9), and GII.2[P16] (*n* = 6) (Table 2). GII.4 Sydney/2012 was the only genotype detected in the serum samples sequenced (Table 2) with identical homology to their paired stool samples (Figure 5). Most of the GII.4 Sydney/2012 sequences clustered with the GII.4[P16] virus detected in Brazil and India in 2018 as well as the other GII.4 Sydney[P16]. The same was observed for the serum samples.

## 4. Discussion

Rotavirus antigenemia among pediatric patients has been well described [21,22,23]. However, this is the first NoV antigenic study of children in Japan. In this study, we found ~55% of children had NoV antigen in their sera (in Mie, Japan); this is consistent with reports from a rotavirus antigenemia study conducted elsewhere [21]. We were also able to detect NoV-RNA in 14.5% of sera screened, a rate higher than that reported in Finland (6.3%) [16] and lower than that reported in Brazil (20.4%) [24]. Of interest, our data suggest a higher prevalence of antigenemia than RNAemia, which may indicate the presence of infectious viruses in the blood.

It was observed that the C_t_ values detected in the sera of patients were significantly higher than those from stool samples (Figure 1A), similar to other study reports [13,24]. There was, however, no correlation between the stool C_t_ values and the likelihood of developing RNAemia, similar to that reported by Takanashi et al. [13]. Nonetheless, a study conducted in Brazil reported a positive correlation between stool C_t_ values and the likelihood of developing RNAemia [24].

It was reported that rotavirus antigenemia is inversely associated with baseline titers of serum rotaviral IgG [25]. Our results also show norovirus antigenemia to be negatively correlated with GII.4 acute antibody titers. Antigenemia was less likely to be detected in children with higher antibody titers; this might be due to the binding of the serum antibodies to the norovirus antigens present in blood and therefore precluding their detection by ELISA, or the serum antibodies may have prevented norovirus antigens from entering the blood.

One of our objectives was to assess the correlation of pre-existing serum IgG levels and the ability of antibodies to cross-react with VLPs of GII.17 Kawasaki-308 and the predominant GII.4 genotype. The majority of the children’s sera tested in this study recognized both GII.4 and GII.17 Kawasaki-308 VLPs using ELISA, with the level of responses more pronounced for GII.4. We also observed no differences in pre-existing GII.4-specific antibody responses when the IgG titers in patients infected with GII.4 Sydney/2012 were compared with those of patients with other GII/GI.2 infections. The low response to the GII.17 variant might be due to a lower circulation of the genotype in the population [26].

The children infected with NoV in this study were stratified into four age groups: 0–2, 3–5, 6–12, and 13–18 years. Those within the 0–2-year age group had the lowest pre-existing antibody levels. Studies have shown that IgG responses during the first year of life are relatively weak and short-lived; therefore, those in the 0–2 age group are more prone to multiple infections [27,28].

This study also sought to determine whether children with antigenemia presented with more severe clinical features of gastroenteritis than children without. We did not find any significant difference in clinical symptoms or disease severity. However, our laboratory results suggest that CRP levels were higher in those without antigenemia, which we could not explain. We also observed that antigenemia was more common in the younger age group (Table 2), which is likely to be associated with the lower antibody titer.

As observed in other studies, the partial genotyping of the positive samples showed a high detection rate of GII.4 variants [24,29]. The second most common circulating genotype was GII.3, which was detected at low frequency at schools in Shanghai, China [30]. The recombinant strains identified in this study have been well documented [31,32,33]. The GII.P16 polymerases were found to frequently recombine with the GII.4 and GII.2 capsid genotypes. Studies conducted in the past revealed that the P16 sequences that harbor the GII.4 Sydney capsids are almost identical to the P16 polymerase type associated with the GII.2 capsid [4,34]. The GII.4 Sydney/2012 variant identified in this study shared similarities with the GII.4 Sydney/2012 strain detected in Brazil and India in 2018.

This study was limited to only hospitalized children. Furthermore, we were unable to detect the presence of infectious viral particles in the serum.

## 5. Conclusions

Our results show that norovirus antigenemia occurred more often in children under 2 years of age with NoV-associated acute gastroenteritis. The occurrence of antigenemia did not correlate with stool viral load or disease severity. It could be hypothesized that the younger children had NoV-Ags in their serum due to their low pre-existing antibodies. This finding calls for an understanding of the levels of antigenemia and its correlation with extraintestinal infection, as well as vaccination.

## Figures and Tables

**Table 1:** Demographics and clinical features of children with/without the NoV-antigen in serum.

Variable	NoV ELISA Positive Serum *n* = 34	NoV ELISA Negative Serum *n* = 28	*p*-Value
Age range, *n* (%)			<0.001
0−2	28 (82)	9 (32)	
3−5	5 (15)	10 (36)	
6−12	1 (3)	9 (29)	
13–18	0 (0)	1 (4)	
Gender, *n* (%)			0.072
Male	23 (68)	12 (43)	
Female	11 (32)	16 (57)	
Clinical Profile			
Max Number of stools/days, *n* (%)			1.000
≤6	31 (91)	26 (93)	
>6	3 (9)	2 (7)	
Diarrhea duration, mean ± SD	2 ± 1.89	1 ± 1.27	0.265
Max Number of vomit/days, *n* (%)			1.000
≤5	23 (68)	19 (68)	
>5	11 (32)	9 (32)	
Vomit duration, mean ± SD	2 ± 1.11	1 ± 0.81	0.136
Temperature °C, *n* (%)			0.780
≤37.5	10 (29)	7 (25)	
>37.5	24 (71)	21 (75)	
Severity Score; mean ± SD	11 ± 3.5	11 ± 2.5	0.615
Clinical chemistry test			
AST, IU/L; mean ± SD	47 ± 25.8	40 ± 19.1	0.201
ALT, IU/L; mean ± SD	28 ± 23.2	29 ± 32.7	0.435
CRP, mg/dL mean ± SD	0.9 ± 2.3	1.8 ± 2.7	0.041

AST: aspartate transaminase; ALT: alanine aminotransferase; CRP: C-reactive protein.

**Table 2:** NoV genotype distribution in infected children.

Genotype	Number of Patients with NoV Positive in Stool (*n* = 63)	Number of Patients with NoV Positive in Stool and Serum (*n* = 9)
**Capsid**		
GI.2	2 (3.2%)	
GII.4-Sydney/2012	32 (50.8%)	4 (44.4%)
GII.3	15 (23.8%)	
GII.2	7 (11.1%)	
GII.NS	7 (11.1%)	5 (55.6%)
**Capsid/Polymerase, *n* = 44**		
GII.4 Sydney[P16]	12 (27.3%)	
GII.4 Sydney[P31]	11 (25%)	
GII.3[P12]	9 (20.5%)	
GII.2[P16]	6 (13.6%)	
GII.NS	6 (13.6%)	

GII.NS: genotype II not sequenced.

## Data Availability

Sequences submitted in this study are available at NCBI GenBank with the accession numbers; OK103809-OK103830, OK103833-OK103836, and OK103839-OK103840.

## Supplementary Materials

The following supporting information can be downloaded at: https://www.mdpi.com/article/10.3390/v14020173/s1. Figure S1: Summary of cases used in this study for statistical analyses, Table S1: Shows the distribution of Norovirus co-infection with other pathogens, Table S2: Reagents used in the reverse transcription (RT) reaction.
