# Supplementary material for: Molecular Epidemiology of Norovirus (NoV) Infection in Mie Prefecture: The Kinetics of Norovirus Antigenemia in Pediatric Patients
Source: Viruses. 2022 Jan 18;14(2):173. doi: 10.3390/v14020173 (PMC8880472; doi:10.3390/v14020173)
Supplement: Supplementary file 1 [file viruses-14-00173-s001.zip › viruses-1483043-supplementary.pdf]

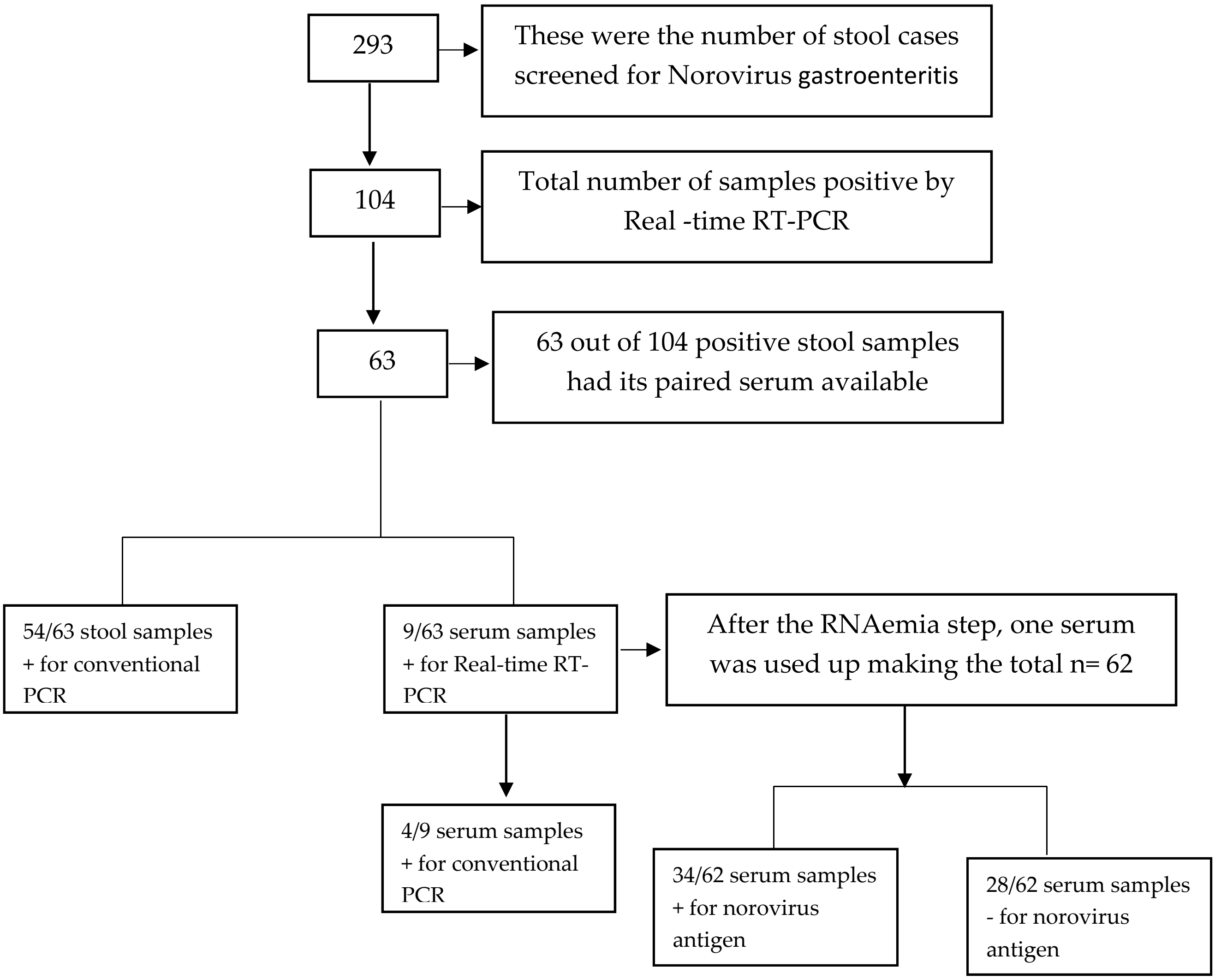

Figure S1. Summary of cases used in this study for statistical analyses

Table S1. Shows the distribution of Norovirus co-infection with other pathogens

Variable	n (%)
Norovirus only	58(92.1)
Norovirus & Adenovirus	1(1.6)
Norovirus & Bacterial agents	4(6.3)

Table S2. Reagents used in the reverse transcription (RT) reaction

RT mix	Volume (µl)
5 X First strand Buffer	3
10mM dNTPs	1
Random Primer	0.5
100mM DTT	1
Ribonuclease Inhibitor	0.67
Super Script II RT	1
DW	2.83
Extracted RNA	10
